# Sex disparity in oronasal presentations of canine transmissible venereal tumour

**DOI:** 10.1002/vetr.1794

**Published:** 2022-07-03

**Authors:** Andrea Strakova, Adrian Baez-Ortega, Jinhong Wang, Elizabeth P. Murchison

**Affiliations:** Transmissible Cancer Group, Department of Veterinary Medicine, University of Cambridge, Cambridge, UK

## Abstract

**Background:**

The canine transmissible venereal tumour (CTVT) is a contagious cancer spread by the direct transfer of living cancer cells. CTVT usually spreads during mating, manifesting as genital tumours. However, oronasal CTVT is also occasionally observed, and presumably arises through oronasal contact with genital CTVT tumours during sniffing and licking.

**Methods:**

Given that sniffing and licking transmission behaviours may differ between sexes, we investigated whether oronasal CTVT shows sex disparity.

**Results:**

Twenty-seven of 32 (84%) primary oronasal tumours in a CTVT tumour database occurred in males. In addition, 53 of 65 (82%) primary oronasal CTVT tumours reported in the published literature involved male hosts. These findings suggest that male dogs are at four to five times greater risk of developing primary oronasal CTVT than females. This disparity may be due to sex differences in licking and sniffing activity, perhaps also influenced by sex differences in CTVT accessibility for these behaviours.

**Conclusion:**

Although oronasal CTVT is rare, it should be considered as a possible diagnosis for oronasal tumours, particularly in male dogs.

## Introduction

The canine transmissible venereal tumour (CTVT) is a contagious cancer affecting dogs. CTVT is a clonal lineage, thus the living cancer cells themselves act as infectious agents and are physically passed between dogs. CTVT originated in a single dog living several thousand years ago,^1–5^ and has since spread through the canine population worldwide.^6^ The disease is prevalent in countries with free-roaming dog populations but also occurs elsewhere in dogs imported from endemic areas.^6,7^

Mating is the most common route of CTVT transmission, and CTVT usually manifests clinically as tumours associated with the external genitalia in both male and female dogs.^8^ However, extragenital body sites, including the skin, eyes, nasal areas, mouth, rectum and internal organs, can also be affected.^6,9–21^ In these instances, if genital involvement co-occurs, the most likely CTVT seeding routes would appear to be either internal metastatic dissemination or self-transmission.^8,20^ The latter could occur, for example, when a dog licks its own genital tumour or makes oronasal contact with its genital tumour while in a curled-up sleeping position. However, in rare cases, primary extragenital CTVT tumours in the absence of genital involvement are observed, which are most likely a result of non-copulatory CTVT transmission. This could occur during activities such as licking, sniffing, scratching or parturition.^9,11–15,22–24^

**Figure 1:**
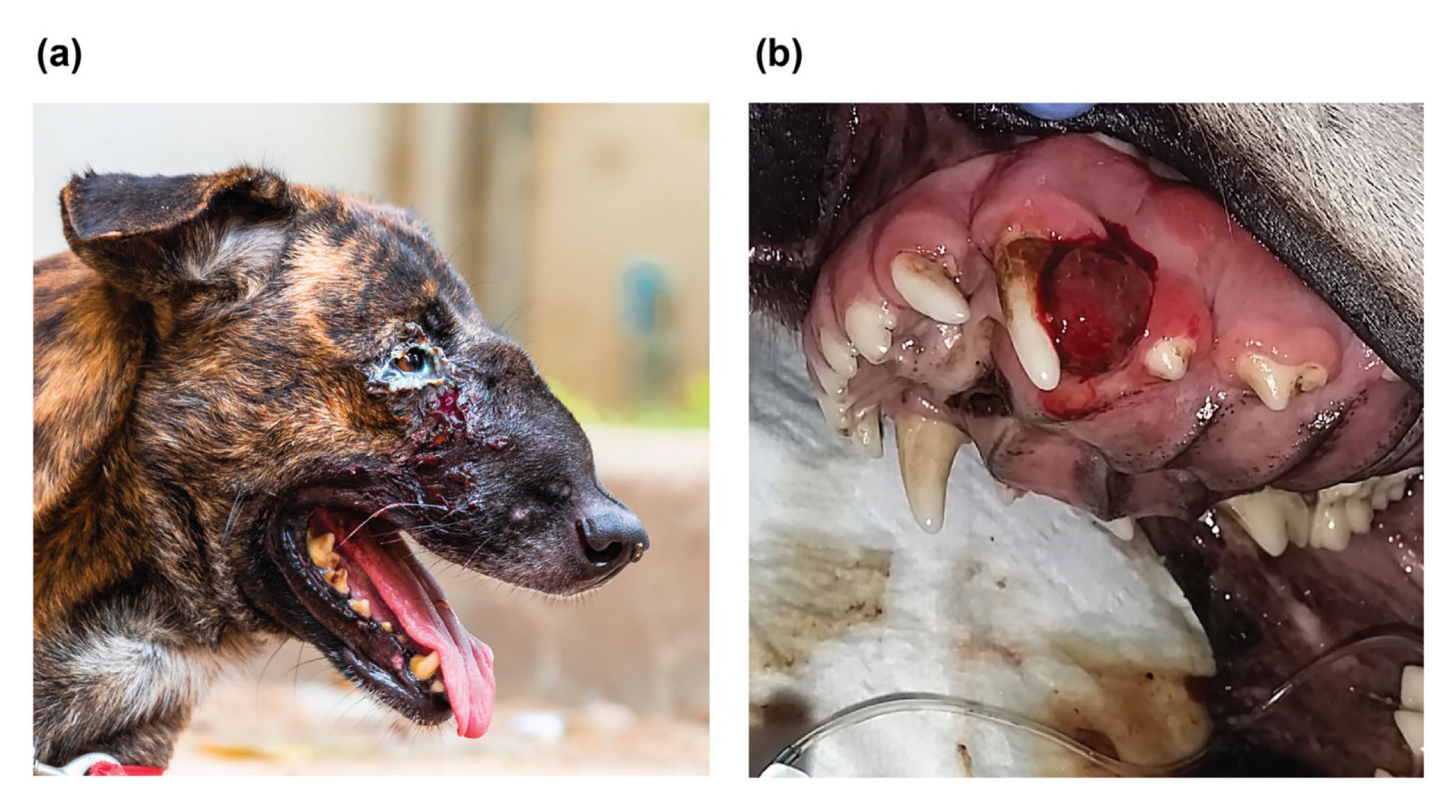
Oronasal presentation of canine transmissible venereal tumour (CTVT). CTVT affecting (a) nasal and (b) oral areas. Photographs provided by Martina Mayr (Animal Rescue Cambodia)/Katherine Polak (FOUR PAWS) and Ada Krupa

One form of extragenital CTVT that often poses a diagnostic challenge is primary oronasal CTVT.^8,22^ Oronasal CTVT is rarely observed,^6,21,22,25^ although to our knowledge no studies have systematically addressed its prevalence. Oronasal CTVT tumours present as soft-tissue masses within the nasal or oral cavities, leading to clinical signs such as sneezing, snoring, mucopurulent nasal discharge or bleeding, and facial deformation^23,26,27^ (Figure 1). Imaging can in some cases reveal paranasal bone destruction.^23,26^

CTVT affects both male and female dogs, and although some studies have reported a higher prevalence in females,^22,28,29^ overall, no consistent sex disparity in CTVT has been reported.^6,30,31^ Variation in the prevalence of oronasal CTVT between the sexes has not been systematically examined; however, there is reason to suspect that there might be sex-linked variation in risk. In females, genital CTVT tumours commonly involve the vulva and are thus more accessible for licking or sniffing than male genital CTVT tumours, which usually occur at the base of the penis and are enclosed within the prepuce. Furthermore, male sensory exploration of female genitals by licking or sniffing may be more common than male same-sex or female activity. In this study, we investigated the hypothesis that primary oronasal CTVT occurs more commonly in male dogs than in female dogs.

## Materials and Methods

This study was approved by the Department of Veterinary Medicine, University of Cambridge, Ethics and Welfare Committee (reference number CR174, 11 November 2015). A database with 1916 records of dogs with confirmed or suspected CTVT tumours was used in this study. The CTVT data were collected between 2009 and 2020 by more than 100 participating veterinarians and other animal health professionals working in first opinion clinical practice, referral clinics or at mass spay/neuter clinics in 59 countries in all inhabited continents. CTVT tumours in the database were considered ‘suspected’ if their diagnosis was based on clinical presentation and clinical history only; ‘confirmed’ CTVT was diagnosed with histopathology, cytology, genetic analysis or a combination of the above.^32–35^ All extragenital CTVT tumours were considered ‘confirmed’. Sex and primary infection site data were available for all 1916 cases. CTVT tumours were classified as genital (defined as primary genital tumours with possible involvement of other sites), oronasal (defined as primary oronasal tumours with no genital involvement) and other extragenital (defined as primary tumours affecting body sites other than the genital or oronasal areas, such as ocular, cutaneous and rectal tumours). Two primary oronasal CTVT tumours had co-occurring tumours involving non-genital body sites (in both cases, eyes).

A literature review was performed in February 2022 by searching for reports of nasal, oral or oronasal CTVT in the published literature using the PubMed and Google Scholar databases. Articles were retained if they reported one or more cases of confirmed primary oronasal CTVT, specified that the relevant animal had no genital involvement, and reported the host dog’s sex. Four primary oronasal CTVT tumours were reported to have co-occurring tumours involving non-genital body sites (eyes, three tumours; rectum, one tumour). The published literature included in the analysis is presented in Supplementary file 1.

Exact binomial tests performed in R^36^ were used to compare the observed numbers of males and females with primary oronasal CTVT in the study database and in the published literature to the 1:1 ratio expected under the null hypothesis.

## Results

In the study database of 1916 dogs with confirmed or suspected CTVT tumours, 1865 had genital involvement. Among the 51 dogs diagnosed with CTVT without genital involvement, 32 had oronasal CTVT, and the remaining 19 had CTVT tumours affecting the eyes, skin, rectum, urethra or inguinal lymph nodes. This corresponds to a 1.7% prevalence of primary oronasal CTVT within this CTVT population (Table 1).

To test our hypothesis that male dogs show a higher prevalence of primary oronasal CTVT than female dogs, we examined the sex of dogs hosting primary oronasal CTVT. Twenty-seven male and five female dogs with primary oronasal CTVT were recorded in the database. Assuming that these animals were drawn from an unbiased source population, this finding provides compelling evidence to discount the null hypothesis that primary oronasal CTVT is equally distributed between the sexes (Table 2) (male proportion = 0.84, 95% confidence interval: 0.67−0.95; *p* = 0.0001, exact binomial test).

A similar sex disparity in primary oronasal CTVT presentation was observed in the published literature. Fifty-three of the 65 cases of primary oronasal CTVT reported in the literature involved male dogs (male proportion = 0.82, 95% confidence interval: 0.7−0.9; *p* = 2.79 × 10^−7^, exact binomial test with the null hypothesis that males and females are equally affected by primary oronasal CTVT) (Table 2, Supplementary file 1).

## Discussion

These data suggest that male dogs are at four to five times greater risk of primary oronasal CTVT than female dogs (Table 2). However, this conclusion relies on the assumption that there was no sex bias in the selection of oronasal CTVT-affected dogs for inclusion in the CTVT database or for inclusion in oronasal CTVT reports in the published literature.

The CTVT database used in this study was compiled from information submitted by more than 100 participating veterinarians and animal health professionals working in 59 countries. Over variable time periods between 2009 and 2020, these participants collected data about dogs with CTVT under their care. Several participants collected data while working in mass spay/neuter campaign settings. Such efforts often prioritise sterilisation of female dogs, and likely as a result of this, the database accessions are, as a whole, biased towards females (1245 females [65%] and 671 males [35%] in the database of 1916 CTVT-affected dogs). This female bias was still observed, although it was less pronounced, in the overall CTVT data provided by participants who contributed one or more primary oronasal CTVT accessions to the database (281 CTVT-affected dogs, 166 females [59%] and 115 males [41%]). We cannot determine if the selection bias that affected the reporting of genital CTVT sex also affected reporting of primary oronasal CTVT sex. It is possible, for instance, that dogs with primary oronasal CTVT came to the attention of participating veterinarians via different routes (e.g., owners directly seeking veterinary care) compared with dogs with genital CTVT (e.g., incidental finding during spay surgery). However, if the female-biased reporting of genital CTVT found in the database applies equally to primary oronasal CTVT, and assuming that there were no biases that caused participants to over-report oronasal CTVT in males, then the true underlying sex disparity in primary oronasal CTVT might be even more pronounced than the four to five-fold increased male risk that we observed.

The finding that primary oronasal CTVT is reported approximately four times more often in male than female dogs in the published literature provides further evidence supporting the notion that male dogs are at greater risk of this form of CTVT. Although it cannot be excluded, we find no reason to believe that authors of published case reviews would be biased towards reporting oronasal CTVT in males. Overall, we believe that the best explanation for the observed disparity in oronasal CTVT prevalence in male and female dogs is an underlying difference in the risk of contracting the disease.

The oronasal form of CTVT is probably transmitted through licking or sniffing genital CTVT tumours or their secretions.^8^ Male dogs recognise oestrous females by sniffing the genitalia,^37,38^ and male dogs sniff vaginal secretion odour more frequently than female dogs.^39,40^ Furthermore, female genital CTVT tumours tend to be more exposed and accessible for licking or sniffing than those of males, which are usually enclosed within the prepuce. Thus, the external location of female genital CTVT tumours, coupled with a likely male preference for licking or sniffing female genitalia, may contribute to increased risk of oronasal CTVT in males. It is not known whether CTVT itself is attractive to dogs; we can only speculate that its odour may mimic oestrus bleeding, which may attract males.^2,41^

Although male dogs appear to be at greater risk of oronasal CTVT, female dogs do also develop this form of CTVT. It follows that, presumably, females also engage in transmission behaviour, including sniffing and licking genitalia of males or other females, but perhaps less frequently than males.

Fewer than 2% of CTVT cases contributed to the study database were of the primary oronasal form (Table 1). Although the inclusion bias in this database, discussed above, precludes estimation of the true proportion of CTVT cases that manifest oronasally, this finding is consistent with reports that primary oronasal CTVT is an unusual presentation of this disease.^6,21,22,25^

The rarity of oronasal CTVT in the population, despite the likelihood that opportunities for licking and sniffing transmission behaviour arise frequently, suggests that transmission of CTVT by sniffing or licking is an unlikely outcome. It is possible that sniffing and licking of CTVT tumours do not usually dislodge cancer cells or that, if dislodged, these cells are unlikely to establish tumours in the recipient’s oral or nasal cavities. CTVT cells have adapted for thousands of years to the genital environment, and oronasal sites are thus likely to be suboptimal for CTVT engraftment. Moreover, oronasal CTVT would appear to be an evolutionary dead end for this contagious cancer lineage, offering limited opportunities for further transmission.

Overall, we report a four to five-fold increased risk of primary oronasal CTVT presentation in male dogs compared with female dogs, highlighting the likely importance of behavioural differences between the sexes in CTVT disease risk when non-copulatory transmission routes are involved. CTVT should be considered as a possible diagnosis for oronasal tumours, especially in male dogs.

## Supplementary Material

Supplementary Table

## Figures and Tables

**Table 1:** Summary of numbers of dogs with confirmed or suspected genital, oronasal and extragenital (other) canine transmissible venereal tumours (CTVTs) in the study database (total number of CTVT cases = 1916)

	Body site of CTVT tumour		
	Genital	Extragenital	
		Oronasal	Extragenital (other)
Number of dogs recorded in the study database	1865 (97.3%)	32 (1.7%)	19 (1%)

*Note*: Dogs with more than one CTVT tumour were included in the ‘genital’ category if one or more tumours involved the genitals, regardless of the body sites of additional tumours. ‘Extragenital (other)’ includes CTVT tumours affecting eyes, skin, rectum, urethra or inguinal lymph nodes.

**Table 2:** Oronasal presentation of canine transmissible venereal tumour (CTVT) is more common in male dogs than in female dogs

	Oronasal CTVT presentation			
	Male	Female	Male:female ratio	*p*-Value
Study database	27 (84%)	5 (16%)	5.4:1	0.0001
Published literature	53 (82%)	12 (18%)	4.4:1	2.79 × 10^−^^7^

*Note*: Exact binomial tests were used to compare the observed number of males and females with primary oronasal CTVT to the 1:1 ratio expected under the null hypothesis of no difference between sexes.

## Data Availability

The data that support the findings of this study are available in the supporting information of this article.
